# Medicine

**Published:** 1899-06

**Authors:** Albert T. Lytle

**Affiliations:** Buffalo. N. Y.


### · MEDICINE.

CONDUCTED BY ALBERT T. LYTLE, M. D., Buffalo, N. Y.

### HYDROCHLORIC ACID IN RHEUMATISM.

R. A. BAYLISS (*British Med. Jour.*, July, 1977,) uses the stronger hydrochloric acid of the British pharmacopeia, applied with a glass brush, for relief of the tenderness in sciatica and pain in the heels and soles in acute rheumatism. The application is made every night or every other night, after which the part is encased in cotton, loosely bandaged. The application does not cause blistering nor does it produce pain. He paints the acid over the tender spots that are located in the thigh and calf. His statistics comprise 16 cases sciatica, 3 not relieved, 11 considerably relieved and two cured; 10 cases plantar and heel pain in rheumatism, 5 not relieved, 1 greatly relieved and 4 cured. The treatment lasted one to five weeks.

---

### TREATMENT OF DIABETES MELLITUS.

F. A. MOUCKTON (*British Med. Jour.*, 1977,) suggests the use of boric acid and sodium sulphocarbolate in the treatment of diabetes mellitus to check the fermentation changes that produce the sugar in the system. He reports successful treatment of several cases.

---

### FUNCTIONAL HEART MURMURS.

DR. MAUDE ABBOTT, (*Phil. Med. Jour.*, 49), in a paper on functional heart murmurs, read before the Montreal Medico-Chirurgical Society, concludes as follows from 466 cases occurring in a list of 2,780 cases examined that: (*a*) Functional murmurs often occur associated with neither anemia or fever, but with some form of intoxication; (*b*) diastolic murmurs have been noted which do not appear to have an organic origin; (*c*) in cases of anemia, pulmonary accentuation is often associated with a pure accidental murmur; (*d*) although accidental murmurs are generally heard at the base and those of relative mitral insufficiency at the apex, accidental murmurs are sometimes, though not always, heard best at the apex.

---

### REACTION OF QUININE ON THE URINE.

CHRISTOMANOS (*Berlin Klinische Wochen.*, October 31, 1898,) calls attention to the fact that quinine in the urine will give a reaction like albumin when the latter is tested for by a solution of picric acid. The heat and the ferrocyanid tests, however, do not give this reaction, but if both albumin and quinine be present in the urine the quantitive determination of albumin by the use of picric acid will give too great a reading.

---

### CASTS IN THE URINE.

A. R. EDWARDS (*Am. Jour. Med. Sciences*, October, 1898,) states that casts in the urine are found more constantly than albumin in cases of nephritis; they may in a given case indicate kidney degeneration rather than inflammation; they are not invariable in nephritis, but they are invariably nephritic.

---

### PYROSAL IN ACUTE RHEUMATISM.

PYROSAL, (*Deut. Med. Woch.*, October, 1898,) a compound containing antipyrin 50 per cent., salicylic acid 36 per cent., acetic acid 14 per cent., dissolving with difficulty in water or alcohol and decomposing readily by the action of acids or bases into its constituents is recommended for use in acute rheumatism.

Phenosal, a compound containing phenacetin 57 per cent. and salicylic acid 43 per cent. acts in a similar manner to pyrosal. The claim is made that upon the whole they are more prompt in their action than salicylic acid alone. Dosage, 0.50 to 0.60 gms, taking not more than 3.50 gms in 24 hours.

---

### INCISION FOR FOOT BLISTERS.

FOOT blisters (*Jour. Am. Med. Assn.*, November, 1898,) heal rapidly without pain or inflammation if they are incised the whole length with a bistoury and emptied, then each lip raised with a sound and the cavity sprinkled with iodoform powder. The blister is then covered with a thin layer of cotton and a small piece of adhesive plaster applied outside. No inconvenience is felt in walking, even at once; the wound dries in a few hours and new epidermis soon forms.

### THE MANAGEMENT OF PATIENTS BEFORE AND AFTER LAPARATOMY.

Dr. Frederick Holme Wiggin (*New York Medical Journal,*) says "if the operation is to be performed at an early morning hour (8 a. m.) the patient should be given a peptonised milk punch at eleven o'clock the previous evening, and if he awakens at 5 or 6 a. m. one ounce of liquid peptonoids may be given, but nothing more. It has been the writer's experience that a small amount—one ounce— of stimulating and concentrated food, administered about two hours prior to taking of the anesthetic, dimishes the liability to heart failure, and also lessens the nausea and vomiting which frequently follow the return to consciousness. If an afternoon hour has been decided upon, another peptonised milk punch may be given the patient when he awakens, and one ounce of liquid peptonoids at 11 o'clock a. m."

In considering the after-treatment, the author says:

"With the passing of these first twelve or eighteen hours, if the patient is not suffering from nausea or vomiting, and the pulse rate is much the same as before the operation, a drachm of liquid peptonoids or some other similar preparation may be given, and repeated, if well borne, every twenty minutes until four doses have been taken, when after an interval of two hours a small quantity of equal parts of milk and lime water or, if peptonised, milk may be given from time to time, until four ounces has been taken. After this there should be an interval of two hours and then half an ounce of liquid peptonoids may be administered."

